# The beneficial effects of different types of exercise interventions on motor and cognitive functions in older age: a systematic review

**DOI:** 10.1186/s11556-017-0189-z

**Published:** 2017-12-21

**Authors:** Oron Levin, Yael Netz, Gal Ziv

**Affiliations:** 1 Movement Control and Neuroplasticity Research Group, Biomedical Sciences Group, Department of Movement Sciences, KU Leuven, Heverlee, Belgium; 2 The Academic College at Wingate, Netanya, Israel

**Keywords:** Exercise, Motor functions, Cognitive functions, Cognitive-motor training, Brain

## Abstract

The decline in cognitive and motor functions with age affects the performance of the aging healthy population in many daily life activities. Physical activity appears to mitigate this decline or even improve motor and cognitive abilities in older adults. The current systematic review will focus mainly on behavioral studies that look into the dual effects of different types of physical training (e.g., balance training, aerobic training, strength training, group sports, etc.) on cognitive and motor tasks in older adults with no known cognitive or motor disabilities or disease. Our search retrieved a total of 1095 likely relevant articles, of which 41 were considered for full-text reading and 19 were included in the review after the full-text reading. Overall, observations from the 19 included studies conclude that improvements on both motor and cognitive functions were found, mainly in interventions that adopt physical-cognitive training or combined exercise training. While this finding advocates the use of multimodal exercise training paradigms or interventions to improve cognitive-motor abilities in older adults, the sizeable inconsistency among training protocols and endpoint measures complicates the generalization of this finding.

## Background

Changes in brain structure and function with age can give rise to a wide range of cognitive and motor declines in healthy older adults [1–13]; see reviews [12, 14–17]. Research over the past two decades has provided compelling evidence that these declines can be delayed or even reversed, and that skills can be revived by engaging in different sports activities and maintaining an active lifestyle; see reviews [18–22]. Given the relatively fast rise in the proportion of older adults in Europe and worldwide, finding new approaches or interventions to improve motor and cognitive functioning and promote healthy lifestyle is of importance. The present systematic review aims at providing a summary of research that has been conducted over the last decade and examined specifically the effect of different types of physical exercise training on both cognitive and motor functions.

In healthy older adults, regular physical exercise training has been reported to improve mood [23], relieve anxiety and depression [24], and enhance global cognitive functions such as memory [24–26], attention [24, 27], inhibition [27–33], and processing speed [22, 34]; see reviews [21, 22, 35]. Besides the beneficial impact of physical training on cognition, it has also been shown to improve mobility [29, 36–39], balance [37, 40], and fine upper limb control [41–44]; see reviews [39, 45, 46]. While there has been a growing number of studies evaluating the effects of physical exercise training on cognition in the past decade, the beneficial effects of training on motor functions per se have received less attention. In addition, the effects of physical exercise training on cognitive functions and motor functions have generally been explored separately. This segregation is somewhat surprising, given that motor and cognitive functions share similar brain network systems, and thus are expected to be influenced by parallel neurodegenerative processes in aging.

For example, age-related changes in the structural and functional integrity of prefrontal and basal ganglia substructures have been reported to be associated with a range of cognitive deficits, such as a decline in memory [47], information processing speed [6, 9, 11], and inhibition [4]; see [48, 49] of the involvement of the prefrontal-basal ganglia network in motor and cognitive functioning. Structural changes in the same substructures can also predict a wide range of motor declines, such as poor performance of complex coordination tasks [3, 8], longer action selection times [50], mobility deterioration [5], and balance loss [2]. Nonetheless, a growing body of evidence suggests that general physical training increases gray matter and white matter volume in prefrontal brain networks ([32, 51–55]; see reviews [20, 22]), which are compromised by aging processes to a greater extent than other regions of the brain [10, 56]. However, note that prefrontal contributions to performance declines cannot be isolated from greater distributed gray and white matter loss in the whole brain [1, 57]. Taken together, these observations suggest that physical training could be an effective means to prevent brain atrophy and maintain (or even improve) cognitive and motor abilities in aging.

As physical activity appears to ameliorate cognitive decline in both healthy aging and age-related pathological conditions ([23, 24, 26, 27, 29–31, 37, 58, 59]; for review see [60]), questions emerge as to what extent improvements in cognitive functions predict gains in motor functions, and to what extent different types of exercise training differentially affect cognitive and motor functions. For example, it has been shown that exercise training reduced the need of prefrontal resources of executive function and attention involved in challenging treadmill walking. This, in turn, was speculated to allow older adults to allocate more attentional resources to processes related to balance control [27]. An alternative working hypothesis, nonetheless, would assume bilateral positive impacts of physical exercise training on both cognitive and motor functioning. Along these lines, the first aim of the current systematic review was to examine the specific beneficial effects of physical exercise interventions on cognitive and motor functioning in healthy older adult population. The second aim was to examine the interplay between cognitive and motor gains in relation to the physical exercise training used. In line with the aforementioned aims, our search strategy predominantly included search combinations of *(i)* common exercise interventions or training protocols such as cardiovascular (aerobic), strength and/or balance [18–46], [58, 59] and *(ii)* motor and cognitive tasks which are commonly used for evaluation of brain-behavior relationships in aging studies such as inhibition, reaction time, and balance control [1–17, 47–50]. We primarily focused on executive functions such as processing, attention, inhibition which have been shown crucial for successful performance of both gross and fine motor functioning such as locomotion, balance control, reaction time, and coordination; for review see [15–17].

### Literature search, selection process, data extraction, and quality assessment

A systematic electronic search of the literature was carried out online through PubMed database that was published between January 2007 and December 2016. The search strategy was conducted by using a keyword search of the following terms:(physical activity OR training OR aerobic OR resistance OR strength OR dance OR yoga OR tai chi OR martial art OR qigong OR endurance OR balance OR cycling OR swimming OR running OR jogging OR walking OR cross country) AND ((cognitive OR cognition OR cognitive function* OR executive function* OR attention OR inhibition) AND (motor OR motor skill* OR motor task* OR motor learning OR reaction time (RT))) OR motor-cognitive. The search was conducted with the following additional filters: publication dates (10 years), age (65+ years), and pathology (NOT Parkinson’s NOT stroke NOT Alzheimer NOT cancer NOT lesions* NOT patients NOT injury). A list of references, which included relevant reviews or original studies with no restrictions on study design and age-range, was also scanned for additional bibliography. Only studies published in English were considered.

The following *inclusion criteria* were implemented: *(i)* a longitudinal study design with at least two intervention groups (short-term or acute effect studies were not considered), *(ii)* physical intervention or combined physical and cognitive intervention (dual-task), and *(iii)* combined motor and cognitive outcomes as an endpoint. Studies were excluded if they: *(i)* were study design reports, *(ii)* did not include at least one cognitive function test and at least one motor function test at baseline or post-intervention phases of the study, *(iii)* were non-interventional or *(iv)* did not include at least one comparison group (i.e. single group pre- and post-test design) or cross-sectional study design. There were also restrictions with respect to the mean age (> 65 years old) and health condition of the included population (no reported neurodegenerative diseases, chronic illnesses and/or overt cognitive impairments).

**Fig. 1:**
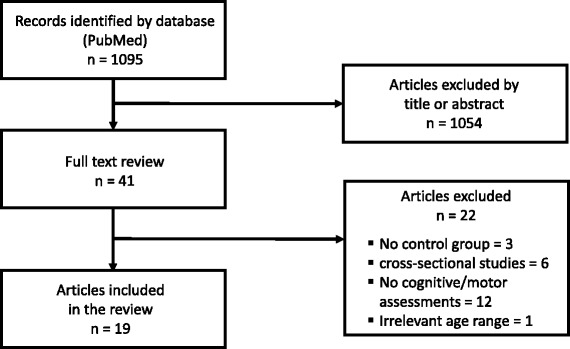
Article selection process

A flow diagram of the study selection process is illustrated in Fig. 1. The search retrieved a total of 1095 likely relevant articles. All retrieved articles were screened by two reviewers (OL and GZ). Doubtful decisions for inclusion/exclusion were resolved by the senior co-author (YN). After screening by title and/or abstract, 1054 articles were excluded due to *(i)* topic irrelevance, *(ii)* being meta-analysis/review papers, *(iii)* irrelevant endpoint outcomes, *(iv)* the inclusion of one or more patients groups, *(v)* being a report of a study protocol with no actual collection of data, and *(vi)* absence of cognitive or motor assessments at baseline and/or endpoint. The remaining 41 articles were evaluated as potentially relevant papers and the full papers were obtained. After screening the full papers, 22 articles were excluded for the following reasons: irrelevant age-range [61], single group pre- and post-test design [62–64], a cross-sectional study design or no exercise intervention [65–70], or absence of cognitive or motor assessments at baseline or endpoint [69, 71–81]. The remaining articles (*n* = 19) met all inclusion criteria and were included in the review [82–100]. Results from the aforementioned 19 articles were summarized with respect to: *(i)* demographic characteristics of participants (total sample size, number of group participants and gender ratio), *(ii)* characteristics of the intervention (exercise protocol, duration/frequency, and exercise intensity), and *(iii)* the outcome effects of the intervention on specific cognitive and motor functions. The aforementioned results are presented in Table 1. Lastly, article quality assessment was conducted using the Jadad scale [101] (see Table 2).

**Table 1:** A Summary of studies (*n* = 19) examining the effect of physical exercise intervention on cognitive and motor functions in older adults

Study (Authors/Year/Ref.)	Intervention	Training protocol	Demographic properties			Endpoint outcomes
			Group	Number of participants/Female	Age Mean (SD) years	
Berryman et al., 2014 [82]	3 groups: Combined aerobic and strength lower body (LBS-A), Combined aerobic and strength upper body (UBS-A) Gross motor activities (GMA).	All groups received training 3 times/week for 8 weeks. LBS-A and UBS-A: 60 min of strength exercises followed by 20-min cycling at 60–65% of max aerobic power. GMA: 60 min exercises of flexibility, walking & ball manipulation.	LBS-A UBS-A GMA	16/9 15/8 16/12	69.8 (3.9) 69.5 (6.1) 72.7 (6.3)	Motor: Significant gains on *TUG*, *chair stands*, *walking speed (10MWT) walking distance (6MWT),* and *grip strength* for LSB-A and USB-A groups. No significant gains for GMA group. Significant group differences in pre/post gains (LBS-A and UBS-A > GMA). Cognitive: Significant gains on both *inhibition* and *working memory* elements of the RNG in dual-task conditions (walking at different speeds). Significant gains on the *inhibition* elements of the RNG in single task condition (rest) for all three groups. No significant group differences in pre/post gains.
de Bruin et al., 2013 [83]	2 groups: Combined motor- cognitive training (M-CG) Motor training alone (MG)	M-CG and MG: 45 min of combined strength & balance training; twice a week for 12 weeks. M-CG: additional 10 min training of divided and selective attention abilities; 3–5 times/week for10 weeks.	M-CG MG	7/5 6/3	> 65^NA^	Motor: Significant decrease in *hand reaction time* for both M-CG and MG but no group differences. M-CG also showed significant decrease in *foot reaction time*. No significant group differences in pre/post gains. Cognitive: Both groups showed significant reduce in *dual task cost* but effects were more prominent for M-CG than for MG. No significant group differences in pre/post gains.
Falbo et al., 2016 [84]	2 groups: Physical single task training (STG) Physical-cognitive dual task training (DTG)	STG and DTG: 60 min of combined physical training (aerobic, balance, resistance, and stretching); twice a week, for 12 weeks. DTG: participants received continuous perceptual-motor cues to adapt their motion patterns during training.	STG DTG	16/14 20/18	73.7 (4.5) 71.5 (6.7)	Motor: Significant gains on *gait stability* for both STG and DTG. No significant changes in *mean gait speed*, *mean stride time*, and *mean stride length*. No significant group differences in pre/post gains. Cognitive: Marginal gain on the *inhibition* elements of the RNG task for DTG. Significant decrease of *inhibitory* performance for STG. No significant group differences in pre/post gains. No significant gains in *dual task cost* for both groups.
Fragala et al., 2014 [85]	2 groups: Resistance exercise (RE) Passive Control (PC).	RG: 7–8 sets for upper and lower limb musculatures at moderate intensity with 8–15 repetition per set; twice a week, for 6 weeks. PCG: No intervention.	RE PC	13^NA^ 12^NA^	70.6 (6.1)^NA^	Motor: Likely beneficial effects (probability >75%) on *reaction time (76%)* and *response time (80%)* and unclear beneficial effect on *movement time* (61%) for RE group. No pre/post differences for PC group. Cognitive: Likely beneficial effect *(85%)* in *spatial awareness* (Neurotracker threshold speed), indicating possible gains on *attention* and *processing speed* for RE group. No pre/post differences for PC group.
Granacher et al., 2010 [86]	2 groups: Balance training (BT) Passive Control (PC).	BTG: 60 min session of postural stabilization tasks (including warmup and cooldown on a bicycle ergometer); 3 sessions per week for 6 weeks. PCG: No intervention.	BT PC	11/7 9/7	72 (5.0) 75 (6.0)	Motor: Significant gains on *gait stability* but not *gait velocity* for BT group in FES-I. No pre/post differences for PC group. Significant Group x Time interaction. Cognitive: Significant gains on *dual task cost* of the secondary motor task but not for cognitive interference task (subtraction mistakes made during the task) for BT group. No pre/post differences for PC group. Significant Group x Time interaction.
Hackney et al., 2015 [87]	2 groups: 90 min Tango classes (TG) 90 min Health education classes (HEG).	Both groups underwent 20 classes over 12 weeks. TG: 20-min standing warm-up followed by 70 min dance exercise. HEG: 90-min seminar classes at local university.	TG HEG	62/48 12/5	82.3 (8.8) 84.1 (7.9)	Motor: Significant gains on *mobility* (Four Square Step, backward gait, and gait speeds) but not *balance* (Berg Balance Scale) for TG. Significant gains on backward gait speed but not for outcome measures for HEG. All gains maintained 3 months after the intervention. Cognitive: Neither group showed any gains on *processing speed*, *working memory*, or *attention*. Cognitive assessments included: Montreal Cognitive Assessment, the Reverse & Corsi Blocks, the Brooks Spatial Task, and the Trail-Making Test (TMT) Part B.
Hamacher et al., 2015 [88]	2 groups: 90 min Dancing session (DG) 90 min Health-related exercise session (HRG).	Both group receive training twice a week for 6 months. Training included 15 min of warm-up and 15 min of cool-down, stretching, and relaxation. DG: learning choreographies of five different dance genres. HRG: 60 min session of combined endurance, strength-endurance, and flexibility training.	DG HRG	19/11 16/10	67.2 (3.4) 68.5 (3.1)	Motor: Both groups showed significant gains on *mobility* but pre/post effects were mixed across group: *(i)* significant increase of gait speed for DG and *(ii)* significant decrease of gait variability for HRG. Main effect for GROUP and the GROUP × TIME interaction were not significant for neither outcome measures. Cognitive: Significant gains on performance of a serial three subtractions test while walking (shorter *successive-subtraction times* and higher *correct answer counts* on) and *dual-task cost* for DG. No pre/post differences for HRG. No significant gains on *dual task cost* for both groups.
Iuliano et al., 2015 [89]	4 groups: Resistance (RG), Cardiovascular (CVG) Postural (PG) Passive control (CG).	Exercises were performed over the course of 12 weeks. RG: 30 min session of high intensity strength training for 6 muscle groups. CVG: 30 min session of high intensity CVG training on ergometer machines PG: 20 min session of flexibility, core-stability and respiratory exercises. CG: No intervention	RG CVG PG CG	20/11 20/12 20/13 20/12	65.8 (6.3) 68.4 (6.4) (66.7) (5.8) 66.5 (6.3)	Motor: Assessment test battery: *(i)* 1MWT for *mobility*, *(ii)* 1RM of shoulder, arm, leg, back, chest, and abdomen muscles for *strength* and *(iii)* SBST for *balance*. RG*:* significantly gains on 1RM test for all six muscles and marginal gains on SBST but not on 1MWT. CVG: significantly gains on 1MWT but not on 1RM and SBST. PG: significant gain on SBST but not on 1MWT and 1RM. CG: no gains on all elements of the test battery. Cognitive: Assessment test battery: AMT, RPMT, Stroop test), TMT, and Drawing Copy Test. RG: significantly gains on Time element of the Drawing Copy Test but not on AMT, RPMT, Stroop test and TMT. CVG: significant gains on all elements of the RPMT and the target element of AMT but not on elements of the Stroop test and TMT. PG and CG: no significant gains on all elements of the cognitive test battery.
Kamegaya et al., 2014 [90]	2 groups: Physical and Leisure Activity intervention (INT) Passive control (CG)	INT: a weekly 2-h intervention for 12 weeks. 45 min of combined physical training (muscle-stretching & aerobic exercise) followed by leisure activities such as cooking, handcrafts, and competitive-games. CG: No intervention.	INT CG	26/24^2^ 26/23^2^	73.6^a^ (5.6) 76.2^a^ (6.1)	^2^Post intervention analysis was conducted with 19 participants in INT group and 20 participants in PCG. Motor: Motor assessment included *(i)* grip test for s*trength* and *(ii)* time up-and-go, 5-m maximum walking times test, and functional reach test for *mobility*. No significant gains on all elements of strength and mobility were observed for neither group. Cognitive: Cognitive assessment included the Five-Cog test for evaluation of attention, memory, visuospatial function, language, and reasoning. Significant Group differences on the reasoning ability element, with significant gains observed only for INT group. Both groups showed significant gains on *attention* and *memory* items and the Wechsler Digit Symbol Substitution.
Leon et al., 2015 [91]	3 groups: Physical exercise (PE), Combined physical and cognitive (PE-C) and Passive control (CG)	PE and PE-C received 60 min training sessions twice a week for 12 weeks. PE: Combined physical training (warmup, strength, aerobic, and relaxation). PE-C: physical training (as PE group) combined with psychomotor tasks, dual tasks, and memory tasks. CG: No intervention	PE PE-C CG	46/38 56/51 35/17	72.6 (5.0) 70.5 (5.5) 71.1 (6.2)	Motor: assessment was based on RT and MT measures of the simple element of the VTS. PC-E and PE and PE-C: significant gains on both MT and RT. Pre/post gains were significantly higher for PC-E than PE and CG (all effects: *p* < .01). Note: CG showed significant gain (*p* < .01) on MT in choice but not simple element of VST. Cognitive: assessment was based on the choice RT element of the VTS (=high cognitive involvement) as an indicator for gains on *processing speed*, *attention,* and *dual-task cost*. PC-E: significant gains on RT in both simple and choice elements of VTS. PE: significant gains on RT only in the simple element of VTS. Pre/post gains were significantly higher for PC-E than PE and CG. GC: no significant gains.
Maki et al., 2012 [92]	2 groups: Community-based walking-program (CBW) Health education classes (HEG)	CBW: 90-min intervention once a week for 12 weeks. 30-min exercise period and 60-min group work. Participants in CBW group planned and executed walking events (excursion) with other group members. HEG: received educational lectures on food, nutrition, and oral care.	CBW HEG	75/52^2^ 75/54^2^	71.9^a^ (4.1) 72.0^a^ (3.9)	^2^Post intervention analysis was conducted with 66 participants in CBW group and 67 participants in HEG. Motor: both CBW and HEG showed significant gains on Timed Up and Go and Walking Speed. HEG showed significant gain on Grip Force (all effects: *p* < .001). No significant group differences. Cognitive: Both CBW and HEG showed significant gains on elements of the Dual task test, Delayed recall test, Digit-Symbol Substitution test, and Yamaguchi Kanji-Symbol Substitution Test (all, *p* ≤ .008). Only CBW group improved on Categorical word fluency (*p* = .003).
Marmeleira et al., 2009 [93]	2 groups: Exercise group (EG) Passive control group (CG)	EG: 60 min exercise of physical (aerobic) and cognitive (psychomotor, dual-tasking, problem solving. 3 days a week for 12 weeks. CG: No intervention	EG CG	16/3 16/4	68.4 (6.7) 68.2 (6.5)	Motor: Significant gains on RT, MT, and response time in simulated driving scenarios under single and dual task conditions for EG. No gains on all elements for CG. Between groups differences were found for MT and response time in single task and RT and response time in dual task. Cognitive: Significant gains on speed of visual processing, but not in divided attention, selective attention, or Stroop (neither incongruent, nor interference scores) for EG. No significant gains for CG. Between groups differences were found only for speed of visual processing.
Schoene et al., 2013 [94]	2 groups: Home-based step-training intervention of Dance-Dance Revolution video game (DDR) Passive control (CG)	DDR: Training for a recommended time of 15–20 min, 2–3 times per week for 8 weeks. CG: No intervention	DDR CG	15^NA^ 17^NA^	77.5 (4.5) 78.4 (4.5)	Motor: assessments were based on elements of the PPA test battery for fall risk, CSRT, TUG, 5STS, and ATS. DDR: significant gains on CSRT and *balance control*. No significant gains were observed for *mobility* and *strength*. CG: no significant gains. Cognitive: assessments were based on elements of TMT; parts A and B and *dual task* performance (verbal fluency during TUG). IG: gain on dual-task ability. No significant gains on all elements of TMT. CG: no significant gains.
Smiley-Oyen et al., 2008 [95]	2 groups: Cardiovascular training (CARDIO) Strength, Flexibility & Balance training (FLEX-TONE).	Both groups trained 3 times per week for 10 months. CARDIO: 25–30 min of aerobic training at 45–60% of HRR. FLEX-TONE: 25–30 min of yoga, Tai Chi, Flex bands, free hand weights, and stability balls.	CARDIO FLEX-TONE	28/21 29/20	69.9 (4.6) 70.5 (4.5)	Neurocognitive test battery was administered: *(i)* RT tests (simple, choice & Go-NoGo), *(ii)* Stroop test (Word, Color, and Word– Color conflict), and *(iii)* WCST. Motor: No significant gains on simple/choice *reaction times* for both groups. Cognitive: Significant gain for CARDIO group and marginal gain for the FLEX-TONE group on RT of the Word–Color conflict element of the Stroop test. Significant gain for CARDIO on error scores of the Word–Color conflict element of the Stroop (*p* < .05). Neither group showed significant gains on the other elements of the neurocognitive assessment tests.
Theill et al., 2013 [96]	3 groups: Aerobic-Cognitive (A-CG), Single Cognitive (S-CG), Passive Control (CG).	A-CG and S-CG participated in 1.5-2 h training sessions twice a week for 10 weeks. A-CG: Cognitive test battery followed by a motor-cognitive dual task while walking +40 min aerobic training (60–80% HHR). S-CG: Cognitive test battery, followed by motor-cognitive dual task while sitting.	A-CG S-CG CG	18/F^NA^ 12/F^NA^ 21/F^NA^	72.4 (4.2) 73.3 (6.1) 70.9 (4.8)	Motor: gait performance was assessed under single- and dual-task conditions while performing a working memory task. Both training groups (A-CG and S-CG) showed significant gains on gait step-to-step variability during dual task but not during single task. CG: no gains. Cognitive: test battery consisted of six computer-based tests to assess for: selective attention, paired-associates learning, executive control, reasoning (Standard Progressive Matrices test), memory span, and information processing speed (Wechsler DSST). Both training groups (A-CG and S-CG) showed significant gains on elements of the executive control task and the Paired Associates task (both involve strong elements of *working memory*) but not in other elements of test battery. CG: no gains.
van het Reve & de Bruin, 2014 [97]	2 groups: Stability-Balance training (SB), Stability-Balance-Cognitive training (SB-C).	SB: 30 min progressive resistance training and 10 min balance training, twice a week for 12 weeks. SB-C: Received in addition to SB training a cognitive training, with the CogniPlus [REF] training program 3 times a week for 10 min.	SB SB-C	98/F^NA^ 84/F^NA^	81.9^a^ (6.3) 81.1^a^ (8.3)	Post intervention analysis was conducted with 77 participants in SB group and 74 participants in SB-C group. Motor: Significant gain on performances of *balance*, *gait initiation*, *chair rise, and simple RT* for both groups. No significant group differences were observed. Cognitive: Significant gain on *(i) dual task cost* of step length (in both preferred walking speed and fast walking speed) and *(ii)* dual task costs of the standard deviation of step length for SB-C. Significant gains on *attention* and *processing speed* (as estimated with TMT parts A and B) for both groups. No group differences.
Vaughan et al. 2014 [98]	2 groups: Combined Strength-Aerobic-Balance training (CTG) Passive Control (CG)	CTG: 60-min class session, twice a week, for 16 weeks. Each session included cardiovascular, strength and motor fitness (balance, co-ordination, flexibility and agility) training + warm-up/cool-down routine. CG: received no training but were on a waiting list to attend the 16-week program.	CTG CG	25/25 24/24	69.0 (3.1) 68.8 (3.5)	Motor: Significant gains for CTG on mobility (6MWT, TUG, One-legged Stance test) and hand and foot *simple RT*. Group differences were significant only for simple RT of the right foot. Cognitive: Significant gains for CTG on *dual task cost* and on all elements of cognitive tests (TMT A and B tests, COAST,Word-Interference and Total time measures, and Controlled Oral Word Association Test measures). Group differences were not significant or marginal.
Williamson et al., 2009 [99]	2 Groups: Combined Strength-Aerobic-Balance-Flexibility training (CTG) Successful Aging health education (SAG).	A 12 mounts intervention program for both groups. CTG: Three center-based exercise sessions (40–60 min) per week for the first two mounts. 2 center-based training and 3 or more home-based training per week for the 4 next mounts. 150 min home-based training for the remaining time. SAG: Meeting sessions on health related topics. Weekly meetings for the first 26 weeks followed by monthly meetings.	CTG SAG	50/36 52/36	76.8 (4.4) 78.1 (4.1)	A complete set of endpoint measurements was conducted on 92 participants (44 CTG and 48 SAG). Only neurocognitive outcome measures (DSST, Rey Auditory Verbal Learning Test, Modified MMSE, and Stroop) and correlations between neurocognitive measures and physical performance measures consisted of SPPB for Grip, Chair stand, Balance, Gait Speed were reported. Cognitive: CTG slightly improved on the DSST but group differences were not significant. Correlations between neurocognitive Physical performance gains for CTG: Post intervention DSST scores (in both groups) significantly correlated with improvements on the Short Physical Performance Battery score (r = .3, chair stand score (r = .26, and balance score (r = .21) but not grip force and gait speed. The Stroop test was marginally associated with the balance score (*r* = − .20).
Yamada et al., 2011 [100]	2 Groups: Strength -Balance-Flexibility training combined with Aerobic stepping exercise in single task (ST) or dual task (DT) conditions.	One training session per week for 24 weeks. Both groups received 20 min of moderate-intensity aerobic exercise (seated stepping), 20 min of progressive strength training, and 10 min of flexibility and balance exercises while seated. Participants in DT were asked to perform a verbal fluency task during seated stepping exercise.	ST DT	26/F^NA^ 26/F^NA^	>65 >65	Motor: DT demonstrated significant gains on functional *mobility* tests (10-m gait speed, 10-m walking cadence) as compared to ST. No significant gains on outcome measures of TUG and functional reaching task were observed. Cognitive: DT demonstrated significant gains on *dual task cost* of the cognitive task (counting numbers aloud in reverse order) during the functional test but group differences were not significant. No gains were observed for ST.

^a^Demographic characteristics of trial participants available only after randomization; ^NA^ Information was not available

^2^refers to a smaller number of participants and described in the outcome column

The bold type in the endpoint outcomes column represent the groups

*1MWT* One Mile Walk Test, *1RM* One Repetition Maximum, *5STS* Five Times Sit-Tostand, *6MWT* Six Minutes Walk Test, *10MWT* Ten Minutes Walk Test, *AMT* Attentive Matrices Test, *ATS* Alternate Step Test, *COAST* California Older Adult Stroop Test, *CSRT* Choice Stepping Reaction Time test, *DSST* Digit Symbol Substitution Test, *FES-I* Fall Efficacy International Test, *MMSE* Mini Mental State Examination, *PPA* Physiological Profile Assessment, *MT* Movement Time, *RNG* Random Number Generator Task, *RPMT* Revan’s Progressive Matrices Test, *RT* Reaction Time, *SBST* Stork Balance Stand Test, *SPPB* Short Physical Performance Battery, *TMT* Trail Making Test, *TUG* Time Up & Go test, *VTS* Vienna Test System, *WCST* Wisconsin Card Sort Test

**Table 2:** Study quality assessment score (Jadad scale [101] with modification^a^)

Study	Randomization (max = 2)	Blinding^a^ (max = 2)	Account of all participants (max = 1)	Total (max = 5)
Berryman et al., 2014 [82]	1	0	1	2
de Bruin et al., 2013 [83]	2	1	1	4
Falbo et al., 2016 [84]	1	0	1	2
Fragala et al., 2014 [85]	1	0	0	1
Granacher et al., 2010 [86]	1	0	1	2
Hackney et al., 2015 [87]	0	1	1	2
Hamacher et al., 2015 [88]	2	1	1	4
Iuliano et al., 2015 [89]	2	0	0	2
Kamegaya et al., 2014 [90]	1	0	1	2
Leon et al., 2015 [91]	1	0	1	2
Maki et al., 2012 [92]	1	0	1	2
Marmeleira et al., 2009 [93]	1	0	1	2
Schoene et al., 2013 [94]	2	1	1	4
Smiley-Oyen et al., 2008 [95]	0	1	1	2
Theill et al., 2013 [96]	0	0	1	1
van het Reve & de Bruin, 2014 [97]	1	0	1	2
Vaughan et al. 2014 [98]	2	1	1	4
Williamson et al., 2009 [99]	2	1	1	4
Yamada et al., 2011 [100]	2	1	1	4

^a^Since participants cannot be blinded to an exercise intervention, a single-blinded study was awarded 1 point despite the fact that the original JADAD scale require double-blinding in order to receive any point

## Results

### Sample characteristics

The number of participants, mean age, and gender distribution for each intervention group in the 19 included studies are summarized in Table 1. Five studies had small sample sizes (*N* < 15) in one or more groups [83]: 2 groups, *N* ≤ 7 per group; [85]: 2 groups, *N* ≤ 13 per group; [86]: 2 groups, *N* ≤ 11 per group; [87]: control group, *N* = 13; [96]: physical-cognitive training group, *N* = 12). In most of the included studies sample sizes per group were larger than 15, and in three studies sample sizes per group were equal to or larger than 50 [92, 97, 99]. In all studies the number of females was larger than that of males, however information about gender distribution within each intervention group was not always available. In one study [98], all of the included participants were female. Subject ages ranged from 55 to 97 years old and mean group ages ranged from 65.5 ± 6.3 [89] to 81.9 ± 6.3 years old [97].

### Interventions

Studies included in this review reported multiple outcome measures, and an extensive range and diverse types of intervention protocols. The most frequent intervention protocol (11 of the 19 included studies) was combined exercise training (e.g. aerobic training followed by resistance training) [82–84, 88, 90, 91, 97–100]. The second most frequent intervention protocol (9 of 19 included studies) was combined physical-cognitive training. Here physical exercise training was either conducted simultaneously with a cognitive task in a dual-task manner [84, 91, 93, 94, 96, 100], or was followed by separate cognitive interventions [83, 92, 97]. The remaining intervention protocols consisted of single-exercise training paradigms, involving aerobic training [89, 95], resistance training [85, 89], balance training [86, 89] or dance [87, 88]. Nine studies included a passive control group [85, 86, 89–91, 93, 94, 96, 98]. Alternatively, participants in control groups underwent health education classes [87, 92, 99] or were subjected to lesser physical (or cognitive) training, for example training of gross motor activities [82] or training of a single cognitive task [96].

Types and durations of the interventions varied considerably between studies. The durations of the intervention period varied, ranging from 6 weeks [85, 86] to 12 months [99]. In most studies the intervention lasted 8 to 12 weeks and consisted of 24 training sessions (or classes) in total. Exercise protocols also varied greatly between studies. For example, the intensity of the aerobic exercise varied from light (e.g. [94]) to moderate-high (e.g. [89]). Durations of the training sessions (for all types of interventions) were inconsistent as well, ranging from 15 to 20 min [89] for balance training to 60–70 min [87, 88] for dance. Similar to the differences in exercise intensity and duration, the type and combinations of exercises varied greatly between studies. For example, three studies that included a combined-exercise training consisted of aerobic-strength training [82, 88, 91], and three studies consisted of strength-balance training [83, 97, 100], whereas in five studies all three exercise paradigms were used in a single training session [85, 88, 90, 98, 99]. Finally, six studies that combined physical-cognitive intervention protocols consisted of physical exercise training with a dual task [84, 91, 93, 94, 96, 100], whereas the interventions in the remaining studies were made up of separate blocks of physical exercise and cognitive training [83, 97] or involved social interactions [92]. Exercises in the physical-cognitive intervention consisted of aerobic training [92–94, 96, 100] or combined aerobic/strength/balance training [83, 84, 91, 97].

### Main outcome measures

Due to the large heterogeneity in exercise protocols and testing methods, it was difficult to arrive at a synthesis of the search findings. Therefore, we performed a descriptive analysis where performance gains (or negative effects) were sorted and summed according to four motor outcome measures and five cognitive outcome measures. The four motor outcome measures were: functional lower limb mobility and gait characteristics [82–84, 86–90, 92–94, 96–100], static and/or dynamic balance [86, 87, 89, 94, 97–99], muscle strength [82, 85, 89, 90, 92, 99], and psychomotor (RT) tasks [83, 85, 91, 93–95, 97, 98]. The five cognitive outcome measures were: processing speed [85–99], working memory [82, 84, 88, 90, 92, 95, 96, 98, 99], inhibition [82, 84, 89, 93–96, 98, 99], attention [85, 87–99], and dual-task cost [82–84, 86, 88, 92–94, 96, 97, 100]. Other outcome measures were aerobic fitness [82, 95], depression scores [87, 90, 92], quality-of-life and life-satisfaction scores [87, 90, 92], and markers of brain plasticity (brain-derived neurotrophic factor – BDNF) [85, 98]. Battery of tests used for the assessments of the aforementioned motor/cognitive outcome measures in each of the included studies are specified in Table 1.

**Fig. 2:**
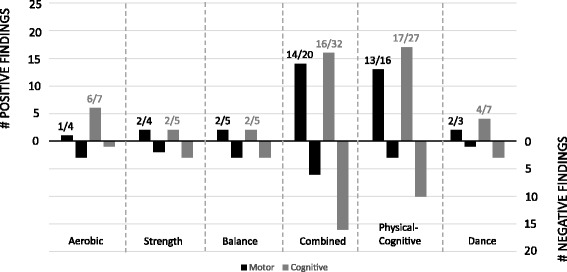
Outcome effects of each of the six types of interventions on overall motor and cognitive functions. Positive findings refer to significant pre-to-post improvements of performance in one or more of the four main motor outcome measures (i.e. mobility, strength, balance, and psychomotor speed) and one or more of the five main cognitive outcome measures (i.e., attention, processing speed, memory, inhibition, and dual-task cost). Negative findings indicate the number of incidences where no significant gains on the abovementioned outcome measures were found. For specific performance gains see Fig. 3 (motor) and Fig. 4 (cognitive)

**Fig. 3:**
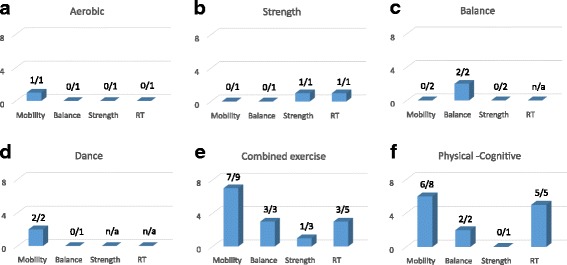
Outcome effects of each of the six types of interventions on motor performance gains (from the number of studies). Please note that one study can measure more than one outcome. **a** aerobic, **b** strength, **c** balance, **d** dance, **e** combined exercise, **f** physical-cognitive. n/a = information was not available

**Fig. 4:**
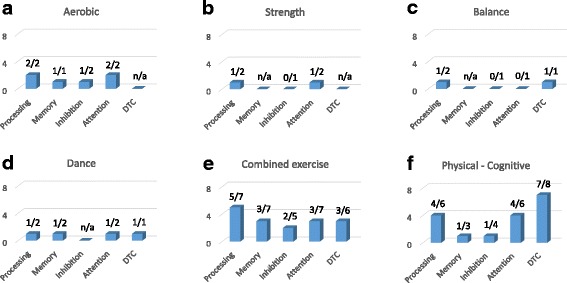
Outcome effects of each of the six types of interventions on cognitive performance gains (from the number of studies). Please note that one study can measure more than one outcome. **a** aerobic, **b** strength, **c** balance, **d** dance, **e** combined exercise, **f** physical-cognitive. n/a = information was not available

The outcome effects of each intervention on specific cognitive and motor functions are presented in Table 1. Data are summarized in Fig. 2 for the overall motor/cognitive gains in each intervention category, and in Figs. 3 and 4 for the specific motor (Fig.3) and cognitive (Fig. 4) gains in each intervention category. As can be seen in Fig. 2, the highest number of reported performance gains and negative findings were reported for the combined exercise training and cognitive-motor training. However, the abovementioned interventions were also the most frequent (Table 1). In line with the first main objective of the current systematic review, the specific effects of the different intervention categories on motor and cognitive gains are described in detail next.

### Motor performance gains as a function of intervention

Motor performance gains (from a number of studies) are illustrated in Fig. 3 for each of the six interventions. The majority of test batteries (or protocols) examined gains in functional tasks (i.e. mobility and strength) [82, 84, 86–90, 92, 94, 97–100], gross motor skills (i.e. balance) [84, 86–89, 94, 96–99], or RT [83, 91, 93–95, 97, 98]. None of the included studies examined fine motor skills or motor learning. Four studies used aerobic [89, 95], strength [85, 89],or balance [86, 89] training as a single intervention. In one study [89], the three interventions and a passive control group were included in a single study design (see Table 1; [89]). Significant gains induced by aerobic training were found only for a mobility pre/post-test (gait speed, One Mile Walk Test), significant gains induced by resistance training were found for the strength pre/post-tests (One Repetition Maximum test in all trained muscles), and significant positive gains induced by balance training have been shown only for a balance pre/post-test (Stork Balance Stand Test). No gains on all elements of the test battery were observed in the passive control group. For the remaining studies, aerobic training [95], resistance training [85], or balance training [86] were applied as single interventions. Likely beneficial gains in performance of the visuomotor RT task (i.e. shorter responses times at likelihood of 80.2%) were reported in [85] and significant gains in gait stability were reported in [86]. Taken together, observations from the four studies suggest that using aerobic [89, 95], strength [85, 89], or balance [86, 89] training as a single intervention may have only limited effects on motor performance gains. However, findings cannot be generalized due to limitations caused by the small number of studies or the diversity in testing protocols – specifically, no inclusion of psychomotor tests [86, 89] and no inclusion of mobility, balance, and strength tests [85, 95].

Two studies focused on dance as a single exercise [87, 88]. Observations from these studies are summarized in Fig. 3D. Irrespective of differences in the intervention and testing protocols, both studies showed a significant increase in gait speed. One of these studies also reported a significant increase in backward gait speed as well as faster performance time on the Four-Square Step Test [87].

Eleven studies used combined-exercise training protocols: (i) aerobic and strength [82, 88, 91], (ii) aerobic and flexibility [90], (iii) strength and balance [83, 95, 97], or (iv) aerobic, strength, and balance exercises combined [84, 88, 98–100]. Observations from these studies are summarized in Fig. 3E. In all studies but two [91, 95], mobility tests were applied pre- and post-intervention, and in seven of nine studies significant gains were observed in one or more mobility performance tests: Time Up & Go (TUG) [82, 98], Walking Speed [82, 97–100], Stride Length Variability [88], and Chair Stand [82, 97, 99]. Improvements in mobility characteristics were observed for all training protocols in which strength exercises were included [82, 88, 97–100], albeit pre-to-post gains in strength were reported only by one study [82] in which two intervention groups and one control group were tested. Three studies reported significant pre-to-post improvements in balance [97–99] and three of four studies reported significant pre-to-post improvements in the performance of one or more psychomotor tests [83, 91, 97]. However, all three studies that reported pre-to-post improvements in balance also included balance training in their intervention. Finally, pre-to-post gains on RT were found in five studies [83, 91, 95, 97, 98]. In two of the studies the training protocol consisted of combined strength and balance exercises [83, 97]. The three remaining studies consisted of aerobic-strength training [91] or aerobic-strength-balance training [98].

Nine intervention studies used one or more paradigms of combined physical-cognitive training. Observations from these studies are summarized in Fig. 3F. In six of the nine studies [84, 91, 93, 94, 96, 100], physical and cognitive training were conducted in a dual-task manner. In the remaining studies [83, 92, 97], participants received the cognitive intervention [83, 97] or social intervention [92] at the end of the physical training. Intervention protocols consisted of: (i) aerobic exercise combined with: a battery of cognitive-psychomotor training [93], memory training [96], a video game [94]; (ii) strength-balance exercise [83, 97] combined with computerized cognitive training for attention; and (iii) aerobic-strength-balance exercise combined with dual-task interference and/or a battery of psychomotor and memory tasks [84, 91, 100]. Most of the pre-to-post performance gains were reported for mobility outcome measures, specifically TUG [92], walking speed [97, 100], stride length/gait speed variability [84, 96], and chair stand [97]. Significant pre-to-post gains were also observed for balance [94, 97]. However, observed gains in the above-mentioned studies were not specific to the intervention, nor to the type of physical exercises or the cognitive training protocols involved. Finally, pre-to-post gains in RT were examined in five studies, in which attention training and/or dual task training exercises were applied [83, 91, 93, 94, 97]. In all five studies a significant improvement in simple RT and/or movement time was observed post-intervention, but significant group differences were evident only when a passive control group was included [91, 93]. Thus, the existence of an evident link between these two types of cognitive training and respective pre-to-post gains in Stepping Reaction Time (SRT) cannot be generalized. To conclude, intervention protocols using single-exercise training tended to result in focal performance gains [86, 89], whereas multiple exercise training [82, 83, 97–99] or physical-cognitive training [83, 94, 97] typically resulted in gains of multiple motor outcome measures.

### Cognitive performance gains as function of intervention

Cognitive performance gains (from the number of studies) are illustrated in Fig. 4 for each of the six interventions. Again, pre-to-post performance gains on cognitive outcome measures were more visible in groups that underwent combined physical exercise training [82–84, 88, 90, 91, 97–100] or combined physical-cognitive training [82, 84, 91–94, 96, 97, 100] than in groups that underwent aerobic training [89, 95], strength training [85, 89] or balance training [86, 89] as a single exercise. Pre-to-post improvements on processing and attention were found in both studies in which aerobic training was applied as a single intervention [89, 95], whereas significant improvements on memory [89] or inhibition [95] were evident only in one of the two studies. Finally, the beneficial effects of strength training [89, 95] or balance training [86, 95] on cognition were marginal, with evidence pointing to possible gains in processing speed [85], attention [89] or motor interference task [86], but not on inhibition [85, 89] or cognitive interference task [86] (see, Figs. 4A-C).

The effects of dance as a single intervention on cognitive functions in older adults were reported in two studies [87, 88]. In one study [87], no pre-to-post gains in cognition were reported. In contrast, the other study [88], which used a cognitive-motor interference task (a serial three subtractions test while walking), found a significant decrease in the average time required to recite the successive subtractions and a marginal increase in the percentage of correct answers, suggesting pre-to-post improvements in processing speed, working memory, attention, and dual-task cost (see, Fig. 4D). Of note, the durations of the single training sessions in both studies were largely similar (90 min including warm-up and cool-down). However, the duration of the intervention was twice as long in one study [88] (26 weeks) than in the other study [87] (12 weeks). This could partially explain the absence of significant post-intervention effects in the latter study.

Findings from the eleven studies in which combined-exercise training protocols were used (see, Fig. 4E) and the nine studies in which combined physical-cognitive training protocols were used (see, Fig. 4F) are discussed next. Due to the large variety among the applied cognitive test batteries, pre-to-post intervention effects are presented for each of the five outcome measures separately, as a function of the different training protocols. Statistically significant pre-to-post-intervention differences on one or more outcome measures of processing were reported for aerobic-flexibility training [90], for strength-balance training [97], and for aerobic-strength-balance [98]. Improvements were found on: (i) Digit Symbol Substitution Test (DSST) and Analogy test scores [90], (ii) TMT parts A and B time scores [97], and (iii) California Older Adult Stroop Test (COAST) and TMT (parts A and B) time scores [98]. Significant pre-to-post-intervention differences on one or more outcome measures of processing were also reported in four studies in which combined physical-cognitive training protocols were used [91–93, 97]. In three of those studies [92, 93, 97], significant pre-to-post improvements on one or more outcome measures of attention or dual-task cost were also found. Significant pre-to-post differences were found: (i) in TMT parts A and B time scores following strength-balance training and computerized attention training [97]; (ii) on the Categorical Word Fluency element of the 5-Cog test, the digit symbol substitution test (DSST), and the Yamaguchi Kanji symbol substitution tests following aerobic training combined with social interaction [92]; (iii) in a visual processing (Useful Field of View Test (UFOV)) [93], and (iv) in the Simple/Choice RT elements of the Vienna Test System [91].

Statistically significant pre-to-post-intervention differences on one or more outcome measures of memory were reported for aerobic-strength training [82] and aerobic-flexibility training [90]. In [82], improvements in two elements of the Random Generator Number test (RNG, R scores, and mean repetition gap (MRG)) were observed only during a dual task (i.e., performing the RNG test during walking), and were more prominent for the intervention group that underwent aerobic training combined with strength exercise of the upper body muscles (UBS-A group) than in the intervention group that underwent strength exercise of the lower body muscles (LBS-A group). Pre-to-post gains on MRG scores of the RNG test were found only for the UBS-A and control groups, but not for the LBS-A. For [90], a significant gain was reported on the Cued Recall Test of the Five-Cog task. However, a comparable improvement was also found in the control group. Pre-to-post-intervention improvements in memory were observed in only one [92] of the three studies [84, 92, 96] where memory tests were conducted after the implementation of combined physical-cognitive training.

Statistically significant pre-to-post-intervention differences on one or more outcome measures of inhibition were reported for aerobic-strength training [82] and aerobic-strength-balance training [98]. In one study [82], significant gains were found for the Turning Point Index (TPI, changes between ascending and descending phases) and the adjacency score (numbers presented in pairs; i.e. 3–4) of the RNG test that were visible in both the single-and dual-task test conditions. However, improvements were not statistically different as a function of the group, and were not consistent across test conditions. With respect to the studies in which combined physical-cognitive training protocols were used, pre-to-post-intervention improvements in the performance of the inhibition component in the RNG test was reported only in one study following an intervention with dual-task walking [84].

Statistically significant pre-to-post-intervention differences on one or more outcome measures of attention were reported for aerobic-flexibility training [90], strength-balance training [97], and aerobic-strength-balance training [98]. Testing protocols were not identical across the three abovementioned studies. For [97], significant pre-to-post improvements were reported in the performance of the divided attention task of the Vienna Test System. For [90], a significant gain was reported on the Character Position Referencing task of the Five-Cog task, but a comparable improvement was also found for participants in the passive control group. Finally, [98] reported significant pre-to-post improvement on performance of the TMT parts A and B tests (see also improvement in processing), but not on the Letter-Number Sequencing task. No significant gains were reported by [95] for strength-balance training and for [88, 99] for aerobic-strength-balance training.

With respect to the studies in which combined physical-cognitive training protocols were used, significant pre-to-post-intervention improvements on one or more outcome measures of attention were reported in four studies [91–93, 97]. With respect to one study [97], significant pre-to-post improvements were also reported on all divided attention elements of the Vienna Test System. Finally, another study [93] reported significant pre-to-post improvement in the divided attention element of the UFOV evaluation tool.

Significant pre-to-post-intervention differences on Dual-Task Cost (DTC) were reported by [82] for aerobic-strength training and [83] for strength-balance training (however, statistical power in the latter study was poor due to the small sample size). In [82], improvements in DTC were associated with improvement in working memory and inhibition, as reported above. Interestingly, for three of the six studies mentioned above [91, 97, 100], significant pre-to-post improvements on DTC were reported when the same physical intervention protocols were repeated whilst cognitive training was added. With respect to the studies in which combined physical-cognitive training protocols were used, pre-to-post-intervention improvements in DTC were reported in seven of the eight studies where this outcome measure was tested [83, 92–94, 96, 97, 100]. Gains were not specific to the intervention program (either to the type of physical exercises or to the cognitive training protocols involved) or to the evaluation protocol.

### Association between motor and cognitive gains

**Fig. 5:**
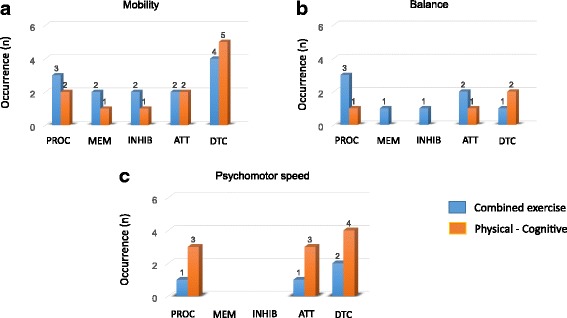
Occurrences of parallel improvements in motor and cognitive outcome measures for combined exercise training (data extracted from *n* = 11 studies) and combined physical-cognitive training (data extracted from *n* = 9 studies). PROC = processing speed; MEM = memory; INHIB = inhibition; ATN = attention; DTC = dual-task cost. Parallel improvements in strength and inhibition were reported only by [82] – data are not shown. See text for the remaining single exercise interventions. **a** mobility, **b** balance, **c** psychomotor speed

In line with the second major aim of the current review– looking into the dual effect of various training protocols on motor and cognition, we provided a qualitative overview of the extent by which pre-to-post gains in motor functions parallel improvements in the performance of cognitive functions. Specifically, a detailed inspection of the data in Table 1 indicates that parallel improvements in motor and cognitive performances were observed, mainly for interventions consisting of combined physical training or combined physical-cognitive training. The occurrences of parallel improvements in motor and cognitive outcome measures are illustrated in Fig. 5 for the two combined training interventions. It can be seen that parallel improvements were mainly found for: *(i)* mobility and dual-task cost (DTC) [82, 83, 92, 96, 97, 99, 100], *(ii)* mobility, balance, processing speed, and attention [97, 98], or *(iii)* psychomotor speed, processing speed, attention, and/or DCT [83, 91, 93, 94, 97]. To a lesser extent, we also found associations between: *(i)* mobility (TUG/gait speed/gait variability), balance and inhibition for physical-cognitive training [84, 98], or *(ii)* between gait speed, strength, and inhibition for combined exercise training [82].

For the remaining interventions (aerobic, strength, balance, and/or dance), parallel improvements in physical/motor outcome measures and cognitive outcome measures were found between: *(i)* mobility (gait speed) and processing speed, attention, memory, and DTC in dance [88]; *(ii)* mobility, attention, and processing speed in aerobic training [89]; *(iii)* psychomotor speed, attention, and processing speed for strength training [85]; and *(iv)* balance, processing speed, and DTC in balance training [86]. Overall, these qualitative analyses suggest that pre-post gains in gait, mobility, and balance were associated with cognitive improvements. However, most of the included studies did not examine correlations between the pre-post difference values of cognitive and motor outcome measures.

Direct assessment of the associations between pre-to-post difference values of cognitive and motor outcome measures were available in only two of the nineteen reviewed studies [84, 99]. One study [84] reported that increased inhibition efficiency was associated with decreased gait variability (*r* = −.65, *p* = .006) in the group that underwent physical-cognitive dual-task training. However, this effect was found only during dual-task walking with simple gait demands. The same authors reported a marginally significant association between the same outcome measures also for the group that underwent the physical training alone (aerobic-strength-balance combined). Here, a significant correlation between increase inhibition efficiency and decreased gait variability (*r* = −.47, *p* = .049) was reported for dual-task walking with complex gait demands (i.e., walking while negotiating hurdles). The second study [99] reported that pre-to-post gains in processing and attention (as measured with DSST) following aerobic-strength-balance training were positively correlated with improvements in the Short Physical Performance Battery (SPPB) scores (*r* = .38, *p* = .002), chair stand scores (*r* = .26, *p* = .012), and to some extent balance scores (*r* = .21, *p* = 047). The same authors also reported significant positive correlations between gains on short-term memory scores (Rey Auditory Verbal Learning Test, (RAVLT)) and gait velocity (*r* = 0.25, *p* = .019) or chair stand (*r* = .22, *p* = .039). Otherwise, correlations between pre-to-post changes in the performance of RAVLT or the inhibition test (Stroop), and pre-to-post changes in all other elements of the SPPB, did not reach the level of significance (*r* ≤ .20, *p* > .05).

## Discussion

In line with the objectives of the current systematic review, the search strategy we used aimed at locating research studies that examined the combined effects of physical training interventions on motor and cognitive functions in older adults. Our literature search and selection process resulted in 19 publications, of which 11 studies reported the effects of combined (multi-component) exercise training [82–84, 88, 90, 91, 97–100], 9 reported the effects of combined physical cognitive training [83, 84, 91–94, 96, 97, 100], and 8 reported the effects of single exercise protocols with aerobic training [89, 95], strength training [85, 89], balance training [86, 89], or dance [87, 88]. The main findings from the 19 included studies were:

1. Multi-component exercise training or combined physical-cognitive training were found to improve a larger number of physical, motor, and cognitive outcome measures than a single exercise intervention. Physical-cognitive training was found to be the best intervention strategy.
2. Multi-component exercise training was found to be beneficial for improving gait and processing speed, whereas combined physical-cognitive training was found to be most beneficial for psychomotor speed, processing speed, attention, and dual task cost.
3. Pre-post gains in mobility and psychomotor speed were strongly associated with pre-to-post gains in processing and dual task cost. However, these associations were more prevalent when intervention consisted of combine physical-cognitive training.
4. Due to lack of sufficient consistency in the training protocols and applied test batteries, we were unable to provide a reliable evaluation of the possible effects of single-exercise protocols on performance.

### Specificity of the exercise interventions

The extent to which exercise interventions were associated with specific gains in cognitive and motor functions were examined in line with the first major aim of the current systematic review. Overall, findings from the nineteen included studies indicated that combined exercise training and physical-cognitive training resulted in significant improvements in mobility (e.g. increased walking speed and reduced time of chair rise), attention, and processing capabilities. Also, improvements in psychomotor speed and dual-tasks cost were more pronounced after physical-cognitive training than after exercise training alone. The aforementioned observations are in line with findings from previous systematic reviews or meta-analysis studies [45, 46], all together providing evidence that multi-component exercise training or combined physical-cognitive training appeared to be the best intervention strategies for improving multiple physical, motor, and cognitive functions. This augmented effect could be attributed to parallel improvements in processing and attention, which were more evident after combined physical-cognitive training than after combined exercise training. Moreover, observations from the nineteen included studies indicated that combined physical-cognitive training had a greater beneficial effect than other types of interventions on processing and attention, but not on inhibition and memory (e.g. [91–93, 97]); partly explaining the observed improvements in the performances of dual-task when this type of intervention was used. However, the above-mentioned findings need to be interpreted with caution, due to the large diversity among the intervention protocols and testing methods.

Pre-to-post improvements in mobility, processing, attention, and/or dual-task cost were also evident in the control group, which underwent physical exercise training or cognitive training as a single intervention (e.g. [95, 96]). The fact that non-significant time × group interactions were observed, indicates that training effects on some of the outcome measures may not necessarily be specific to the training protocol, but may have been caused by merely engaging in physical activity once or twice a week. For example, in five of the nine studies that used combined physical-cognitive training [83, 84, 91, 97, 100], pre-to-post gains in mobility outcome measures were statistically similar to the respective gains found in the control groups that underwent physical training alone. Moreover, the performance gains observed in participants who received the training were not always statistically different from the gains observed in participants in the control groups who attended health education classes [92, 99] or received cognitive training alone [96]. Nonetheless, significant group differences were observed in seven of the nine studies where a passive control group was included [85, 86, 89, 91, 93, 94, 96]. Taken together, one could suggest that adding cognitive elements to the physical intervention may have only a minor additional effect on the mobility characteristics at the post-tests. However, a closer inspection of the findings indicated that improvements in mobility were associated to a greater extent with pre-post gains in dual-task cost after combined physical-cognitive training than after exercise training alone (e.g. [84]). From a brain-behavior perspective, parallel improvements in mobility characteristics and dual-task cost may suggest improvements in the functioning of the basal ganglia and prefrontal cortex [2, 5, 50, 102].

Significant gains in mobility or functional motor tests (e.g., chair rise) could be attributed, at least in part, to significant gains in cardiovascular performance (e.g., [59]; for studies included in the present review see [82, 95]) or increase of muscle strength [29, 31]. The findings from studies included in the current review [84, 98–100] suggest that multimodal combined training would likely lead to greater benefits for general health, cardio-respiratory fitness, and general improvement of cognitive and motor functions than aerobic, strength, or balance training alone. Nonetheless, due to the diversity in interventions and test protocols among the nineteen included studies, we were unable to make a clear association between the types of training used and their specific effects on performance.

### Cognitive-motor interactions

Intervention effects on both cognitive and motor functions were examined, in line with the second major aim of the current systematic review. Findings from the nineteen included studies suggest that intervention effects on mobility, balance, and psychomotor speed were associated with improvements in attention, processing, and dual-tasks (Fig. 5). Intervention effects on mobility and balance, together with improvements in inhibition or memory, were also observed, but were less evident. Importantly, parallel improvements in physical (motor) and cognitive outcome measures were observed in the majority of the intervention groups (75%) that underwent combined physical-cognitive training, but only in about 35% of the groups that underwent physical-exercise training only. This observation suggests, at first sight, that positive training effects (in both motor and cognitive function) might be attributed exclusively to the inclusion of cognitive training; specifically dual-task training [84, 91, 93, 94, 96, 100]; see for further evidence [27, 29, 31]. Taken together, the aforementioned findings suggest that the beneficial effects of physical-cognitive training (in general) and dual task training (in particular) appeared to be superior to other forms of training protocols. However, a closer inspection of the findings indicated that training effects on dual-task performance occurred in parallel to pre-to-post gains in attention, processing, and psychomotor speed (e.g. [97]). Moreover, parallel improvement in physical or cognitive functions under dual-tasks were also evident (albeit to a lesser extent) in studies that used physical exercise training as the main intervention (e.g. [82]), and improvements in physical performance tasks were found when performed under single-task conditions (e.g. [86]). To conclude, the main body of evidence from the current systematic review suggests that combined exercise and cognitive training (in particular when the cognitive training consists of a dual task) could improve basic cognitive and motor functions, and give rise to better management of brain resources [25, 59, 102–104]. This observation is not surprising given the fact that training under a dual task requires sustained attention to visual or auditory stimuli, effective processing of sensory information, and effective transfer of information among the brain’s sensory centers. As such, we expect that this type of intervention would infiltrate high-order executive-control centers and sensorimotor centers, causing neuroplastic changes in widespread areas of the aging brain, as compared to other types of interventions which may induce more local effects. These findings must be interpreted with caution, however, given the low number of included studies and large variety in the intervention and test protocols.

The association between changes in inhibition and mobility or inhibition and balance could be attributed partly to the beneficial effects of cardiovascular training [95]; see for further evidence [103, 105]. However, evidence from other included studies that also applied cardiovascular training either exclusively or solely [89], or in combination with other physical/cognitive exercises [93, 96], failed to support this assumption. Notably, positive training effects on attention could indicate adaptation of a shared attention-inhibition substructure, for example the dorsolateral prefrontal cortex or the prefrontal-basal ganglia network [6, 102, 103]. This could entail selective benefits for attention and inhibition or inhibition and gait performance under a dual task. However, associations between changes in inhibition and attention [98] or inhibition and dual-task walking [84] were rather scarce, as compared to associations between basic cognitive functions and mobility or psychomotor speed (Fig. 5).

Negative findings were reported in the majority (69%) of the included studies where pre-to-post training effects on inhibitory functions were examined [89, 93–96, 99]; see also [84] for combined exercise training but not combined physical-cognitive training. The absence of consistent findings on inhibition across the included studies could be attributed to the large diversity among training protocols, intervention durations, and assessment tools. However, the fact that most of the included studies did report significant training effects when pre-to-post differences were examined for attention (61%), processing (67%), and dual-task costs (75%), implies that some training protocols showed selective beneficial effects for inhibition, where others did not. In line with these observations, we propose that inhibition (and possibly also memory) may be responsive to specific types of training paradigms, whereas other basic cognitive (or motor) functions such as attention or mobility may be responsive to a broader range of interventions or multimodal training protocols. Similar to the findings from two other systematic reviews [18, 20], findings from the current review suggest that multimodal interventions have a greater beneficial effect on older adults than do single interventions – specifically, improving a broader range of cognitive-motor functions and having a better potential protective effect on the structural and functional integrity of the aging brain. Further insights into the effects of specific training protocols on pre-to-post differences in brain-behavior relationships should be considered in future research by including brain imaging techniques.

### Brain-behavior relationships

While evidence from other research studies or systematic reviews could provide some indications about training-induced reorganization of the brain ([26, 32, 51–55]; see reviews [20, 22]), none of the included studies in this review included direct measurements of training-induced differences in brain structure. Indirect evidence for possible relationships between cognitive and motor performance gains and brain plasticity have been examined, nonetheless, in two of the nineteen included studies [85, 98], based on the measurement of brain-derived neurotrophic factor (BDNF) levels in serum or plasma; yet, these observations were inconsistent. Specifically, one study [98] reported a significant increase in the levels of plasma BDNF in response to a combined physical exercise intervention which included cardiovascular, strength, and motor fitness training. The same authors reported a decrease in BDNF levels in participants of the passive control group, which did not receive any exercise training during the period of the intervention. This was also the observation from other studies, where an increased BDNF level in older adults has been reported in response to physical exercise [106, 107], dance [52], or combined physical-cognitive training [106]; see review [20]. In addition, it was found that individuals who experienced greater fitness improvements from the exercise training (i.e., high responders to exercise) also had greater increases in the serum neurotrophic factors, such as BDNF and insulin-like growth factor-1 (IGF-1) [106].

In contrast to the aforementioned observations, one study [85] reported no significant change of serum BDNF in response to a resistance training intervention. Yet evidence also suggests that increases in serum neurotrophic factors appeared to be less responsive to resistance training as compared to other exercise interventions; see [18] for a systematic review and meta-analysis. Furthermore, inconsistencies in findings could be attributed to differences in gender and intensity/duration of the intervention [18, 51, 107] or individual difference in baseline levels of BDNF [18, 108].

Notably, changes in levels BDNF (or other neurotrophic factors) could provide a general indication for neuroplasticity, but cannot provide indications about the exact brain regions or networks that were affected by the intervention. For that reason, studies comparing two or more modes of interventions against a single mode (or controls) should also include pre/post measurements of brain structures. Including neuroimaging data in future studies will be important in order to examine changes in gray or white matter, or brain metabolic profiles to examine the effect of an intervention on neurotransmitter concentration or integrity of brain tissue) at specific brain regions. Some studies have already taken this step [26, 32, 51, 53]. However, most studies reported cross-sectional associations between self-reported physical activity and gray matter or white matter volume (see, for review [20]). Therefore, in future studies, assessments should be made of: *(i)* the extent to which the brain structure and functions are influenced by different types of interventions, and *(ii)* the extent to which brain structural and functional changes occur along with pre-to-post intervention changes in motor and cognitive measurements assessed before and after exercise.

### Limitations

The present systematic review has several limitations. First, the included studies applied very heterogeneous intervention protocols and test batteries, which limited our ability to gain conclusive insights into the specific training effect of each type of intervention – in particular, the lack of consistency among the outcome measures tested in each study and the use of different test batteries for assessment of the same outcome measure. In addition, we found some mismatches between the physical fitness components of training and the reported outcome measures, especially in studies where combined physical-cognitive training protocols were used. For example, nine of eleven studies included strength exercises in the combined-exercise training protocol, but only three studies performed pre- and post-intervention tests of strength [82, 90, 99]. Taken together, this large diversity in methodology hindered our ability to compare results from different studies and perform a quantitative meta-analysis.

Second, all included studies in the current review reported pre-to-post intervention gains on multiple outcome measures. But in none of the included studies were adjustments for multiple testing across dependent variables made; post-hoc comparisons for significant main effects from analyses of variance or covariance, or mixed model regressions within each dependent variable were adjusted (for the most part) by using the Bonferroni correction [82, 84, 86, 89–92, 95]. However, this does not address the issue of Type-1 error for testing across multiple dependent variables; see for example [82, 87, 89, 92–94, 99, 100]. Therefore, we encourage scientists in this area to adapt a more conservative approach for evaluating their findings; for example, to discuss multivariate results at the *p* < .001 level or greater (e.g. [109]) or to apply a false discovery rate procedure [110].

Other limitations may pertain to the use of a single data-base source (PubMed) and/or the elimination of studies which included patient groups. However, PubMed is considered to be a reliable source, and offers free access to most research articles, meta-analysis papers, and systematic reviews. Therefore, it is most likely that very few studies, if at all, may be found in other scientific sources. The inclusion of patient groups may, on the one hand, allow more specific insights into mechanisms or brain structures which may benefit from the intervention. On the other hand, variability among patients regarding the type and severity of their pathological conditions is expected, complicating the synthesis of the search findings.

## Conclusions

Findings from the nineteen included studies indicated that the majority of training effects affected mobility. The same training protocols also appeared to improve attention, processing, and dual-task cost to a greater degree than inhibition and memory. In line with findings from other studies that examined the effects of multimodal combined training on cognitive functions, observations from the studies included in our systematic review indicate that simultaneous training of cognitive and physical abilities has the highest potential to induce simultaneous gains in motor cognitive abilities. Unfortunately, none of the included studies in this review examined a parallel effect of training on brain plasticity, albeit findings from one study [98] reported a significant increase in the levels of plasma BDNF as a result of the intervention. More research is required to determine the exact effects of cognitive-physical training on structural and functional changes in specific brain areas, as well as on interactions between functionally interconnected brain networks. Finally, we encourage scientists in this area to develop specific and consistent test batteries for assessing cognitive and motor effects of exercise. This will enable a clearer picture of the effects of exercise, and will make it possible to conduct reviews and draw general conclusions.

## Abbreviations

10MWT: Ten Minutes Walk Test
1MWT: One Mile Walk Test
1RM: One Repetition Maximum
5STS: Five Times Sit-Tostand
6MWT: Six Minutes Walk Test
AMT: Attentive Matrices Test
ATS: Alternate Step Test
COAST: California Older Adult Stroop Test
CSRT: Choice Stepping Reaction Time test
DNF: Brain-derived neurotrophic factor
DSST: Digit Symbol Substitution Test
DTC: Dual-Task Cost
FES-I: Fall Efficacy International Test
LBS-A: Lower Body Strength & Aerobics
MMSE: Mini Mental State Examination
MRG: Mean Repetition Gap
MT: Movement Time
PPA: Physiological Profile Assessment
RAVLT: Rey Auditory Verbal Learning Test
RNG: Random Number Generator Task
RPMT: Revan’s Progressive Matrices Test
RT: Reaction Time
SBST: Stork Balance Stand Test
SPPB: Short Physical Performance Battery
SRT: Stepping Reaction Time
TMT: Trail Making Test
TUG: Time Up & Go Test
UBS-A: Upper Body Strength & Aerobics
UFOV: Useful Field of View Test
VTS: Vienna Test System
WCST: Wisconsin Card Sort Test
